# High-throughput scaffold-free microtissues through 3D printing

**DOI:** 10.1186/s41205-018-0029-4

**Published:** 2018-11-22

**Authors:** Christen J. Boyer, David H. Ballard, Mansoureh Barzegar, J. Winny Yun, Jennifer E. Woerner, Ghali E. Ghali, Moheb Boktor, Yuping Wang, J. Steven Alexander

**Affiliations:** 1 Molecular and Cellular Physiology, Louisiana State University Health Sciences Center, Shreveport, Louisiana, USA; 2 Oral and Maxillofacial Surgery, Louisiana State University Health Sciences Center, Shreveport, Louisiana, USA; 3 Mallinckrodt Institute of Radiology, Washington University School of Medicine, St Louis, MO USA; 4 Gastroenterology and Hepatology, Louisiana State University Health Sciences Center, Shreveport, Louisiana, USA; 5 Obstetrics and Gynecology, LSU Health Sciences Center, Shreveport, Louisiana, USA

**Keywords:** Microtissues, Spheroids, Screening, 3D printing

## Abstract

**Background:**

Three-dimensional (3D) cell cultures and 3D bioprinting have recently gained attention based on their multiple advantages over two-dimensional (2D) cell cultures, which have less translational potential to recapitulate human physiology. 3D scaffold supports, cell aggregate systems and hydrogels have been shown to accurately mimic native tissues and support more relevant cell-cell interactions for studying effects of drugs and bioactive agents on cells in 3D. The development of cost-effective, high-throughput and scaffold-free microtissue assays remains challenging. In the present study, consumer grade 3D printing was examined as a fabrication method for creation of high-throughput scaffold-free 3D spheroidal microtissues.

**Results:**

Consumer grade 3D printing was capable of forming 96-well cell culture inserts to create scaffold-free microtissues in liquid suspensions. The inserts were seeded with human glioblastoma, placental-derived mesenchymal stem cells, and intestinal smooth muscle cells. These inserts allowed for consistent formation of cell density-controllable microtissues that permit screening of bioactive agents.

**Conclusion:**

A variety of different cell types, co-cultures, and drugs may be evaluated with this 3D printed microtissue insert. It is suggested that the microtissue inserts may benefit 3D cell culture researchers as an economical assay solution with applications in pharmaceuticals, disease modeling, and tissue-engineering.

## Background

Three-dimensional (3D) printing, also known as additive manufacturing, is expected to be a disruptive manufacturing technique and have applications in a variety of future biomedical technologies. The technique involves a bottom-up fabrication, where systems and constructs are created in a layer-by-layer manner. 3D printing has been used for decades and more recently has experienced numerous advancements in speed, resolution, accuracy, cost, and biocompatible materials. Materials that are now compatible with 3D printing include; metals, ceramics, plastics, foods, electronics, biopolymers and living cells [1, 2].

Interest in medical applications of 3D printing is rapidly expanding. Customized surgical tools, guides, implants, prosthetics, and preoperative planning have been used successfully in patient treatment [3–5]. It is thought that customized tissues and organs will also be feasible in the future through 3D bioprinting. 3D bioprinting allows for complex scaffold geometries to be fabricated with desired cell types encapsulated within biomaterials. While the field of 3D bioprinting is still in its infancy, it is experiencing major market growth and holds tremendous potential in tissue engineering, pharmaceutical research, disease modeling, and drug discovery [6].

3D cell cultures have recently gained tremendous attention due to their superiority over 2D cell cultures, which have less translational potential. Cell proliferation, drug uptake, cell morphology, oxygenation, nutrient uptake, waste excretion, and junction protein contents all differ when comparing 3D to 2D cell culture [7]. 3D scaffold supports, cell aggregate systems and hydrogels have been shown to more accurately mimic native tissues and support more relevant cell-cell interactions for studying actions of drugs and bioactive agents [8–12]. 3D cell cultures can be fabricated through a variety of techniques including; 3D bioprinting, low-attachment culture plates, liquid suspension, microfluidics, and magnetic levitation [13, 14]. Here, consumer grade 3D printing was examined as a fabrication method for creation of high-throughput scaffold-free 3D spheroidal microtissues.

## Methods

### 3D microtissue insert design and fabrication

**Fig. 1:**
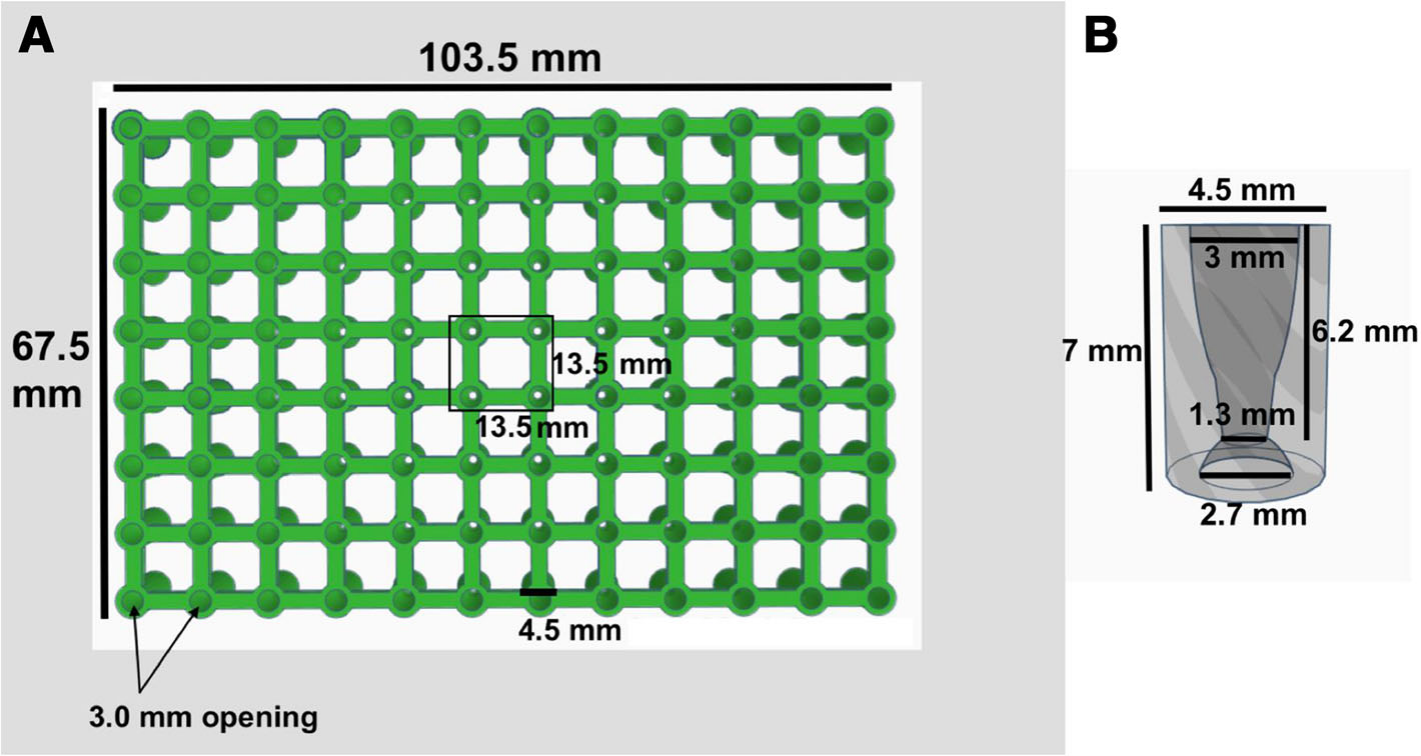
Top view of (**a**) CAD 96 well insert with dimensions displayed and a hollow side view of (**b**) an individual insert with dimensions

**Fig. 2:**
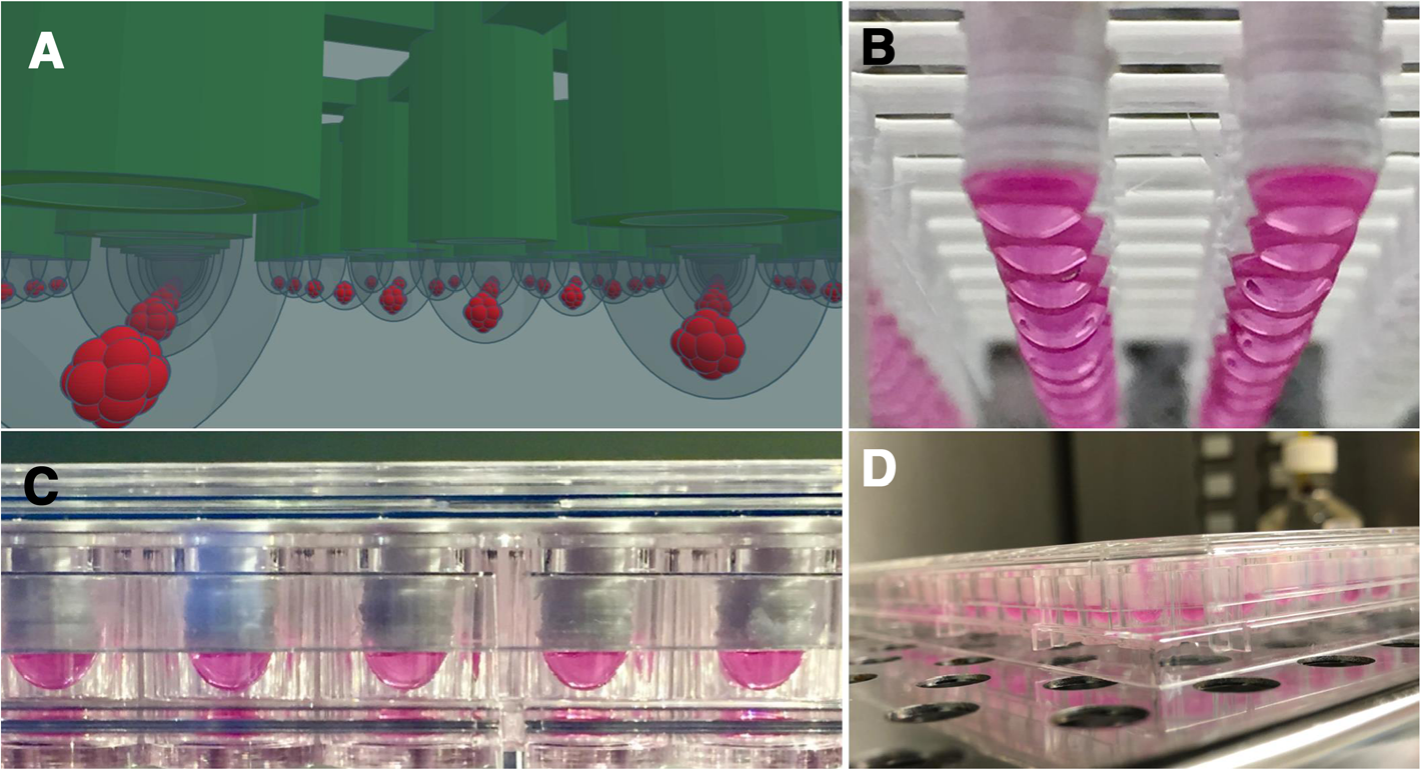
Images of 96-well 3D printed inserts. **a** CAD model and (**b**-**d**) 3D printed inserts with liquid suspensions

Ninety-six well 3D-microtissue inserts were generated using computer-aided design (CAD) software (TinkerCAD, AutoDesk, San Francisco, California). Upper openings of the well inserts were designed with internal tapering to guide pipet tips while the well bottoms were designed with negative hemispherical spacing to hold cell-laden droplets (see Figs. 1, 2). Ninety-six well inserts were 3D printed using polylactic acid (PLA) (PLA-Pro, eSun, Shenzhen, China) at 205 °C on a Lulzbot Taz-6 3D printer (Lulzbot, Aleph Objects, Loveland, Colorado) and were 3D printed in an inverted (180° - upside down) configuration with supports off. Finished 3D printed inserts were removed from the print bed with a spatula and the prints were briefly exposed to a heat gun (~ 200 °C) to remove small flash fibers created during the print process. Additionally, any unwanted larger print defects were manually removed with surgical scissors. Finished 3D printed inserts were submerged in 70% ethanol for 24 h and allowed to air dry over-night in a sterile cell culture hood before beginning cellular experiments.

### 3D microtissue formation and analysis

Three different cell types were examined with the 3D printed inserts. Human placental-derived mesenchymal stem cells (h-PMSC), U87 MG human glioblastoma cells (U87), and human intestinal smooth muscle cells (h-ISMC) were all grown to confluency in flat polystyrene flasks, trypsinized (0.2%/4 mM EDTA), and resuspended in Dulbecco’s Modified Eagle’s medium (DMEM) containing 10% fetal bovine serum (FBS), 1% penicillin/streptomycin (P/S), and 4.5 g glucose/liter (‘insert media’). The 3D printed microtissue inserts were placed in standard flat-bottom 96 well plates and were seeded with 40 μl (μl) of insert media with cells suspended in each drop. The solution pipetting rate was performed slowly to allow droplets to form underneath the 3D printed insert.

Cells seeded in 3D printed inserts were incubated at 37 °C, 7.5% carbon dioxide (CO_2_), and 100% humidity for 72 h (hrs.). Cells were monitored in the 3D printed inserts over the course of 72 h. while in liquid suspension. Cell-loaded 3D printed insert were monitored by both optical and fluorescence microscopy on an EVOS FL Cell Imaging System (ThermoFisher Scientific, Waltham, Massachusetts). 3D spheroids were live-dead labeled with Calcein-AM, Ethidium Homodimer-1, and Hoechst 33342. To demonstrate cell-density dependent spheroid sizing, serial dilutions of h-PMSC (890–14,251 cells) in 40 μl of insert media were injected into the 3D printed inserts and spheroid diameters measured using NIH Image-J software. To measure the spheroid diameter formation, 96 well plates were gently tapped against a flat surface to encourage spheroids droplets to fall to the bottom of the wells. This may also be accomplished by pipetting air through the inserts or by centrifugation.

## Results

**Fig. 3:**
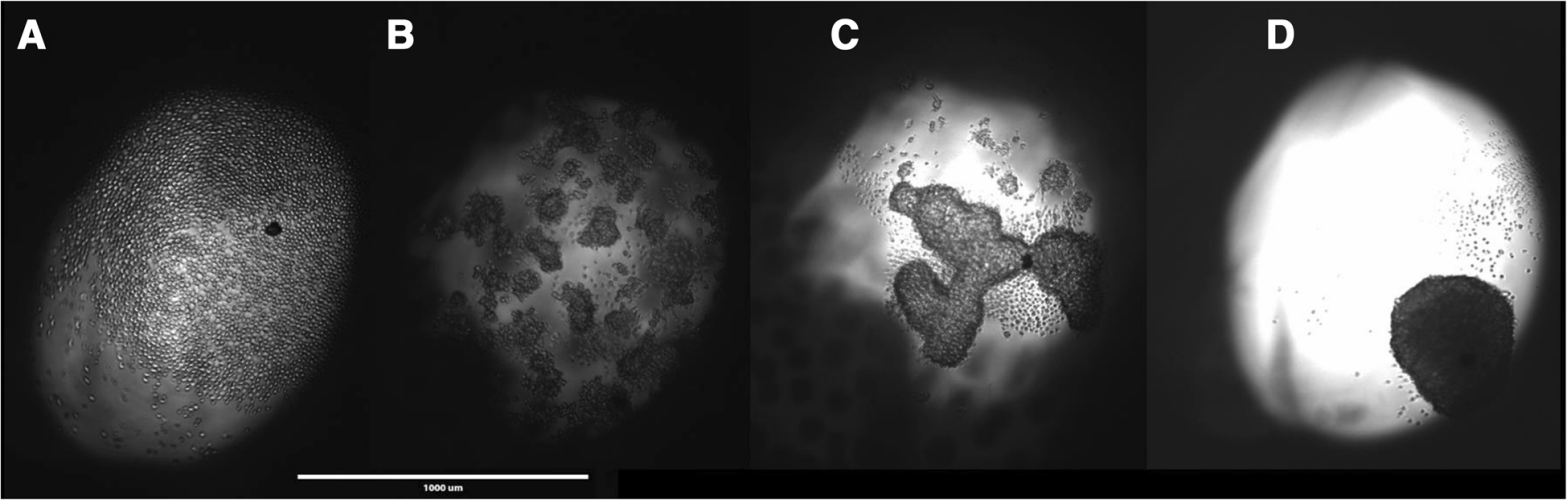
Phase microscopy images of U87 MG human glioblastoma cells at (**a**) 10 min, (**b**) 24 h, (**c**) 48 h, and (**d**) 72 h. Scale bar = 1000 μm

**Fig. 4:**
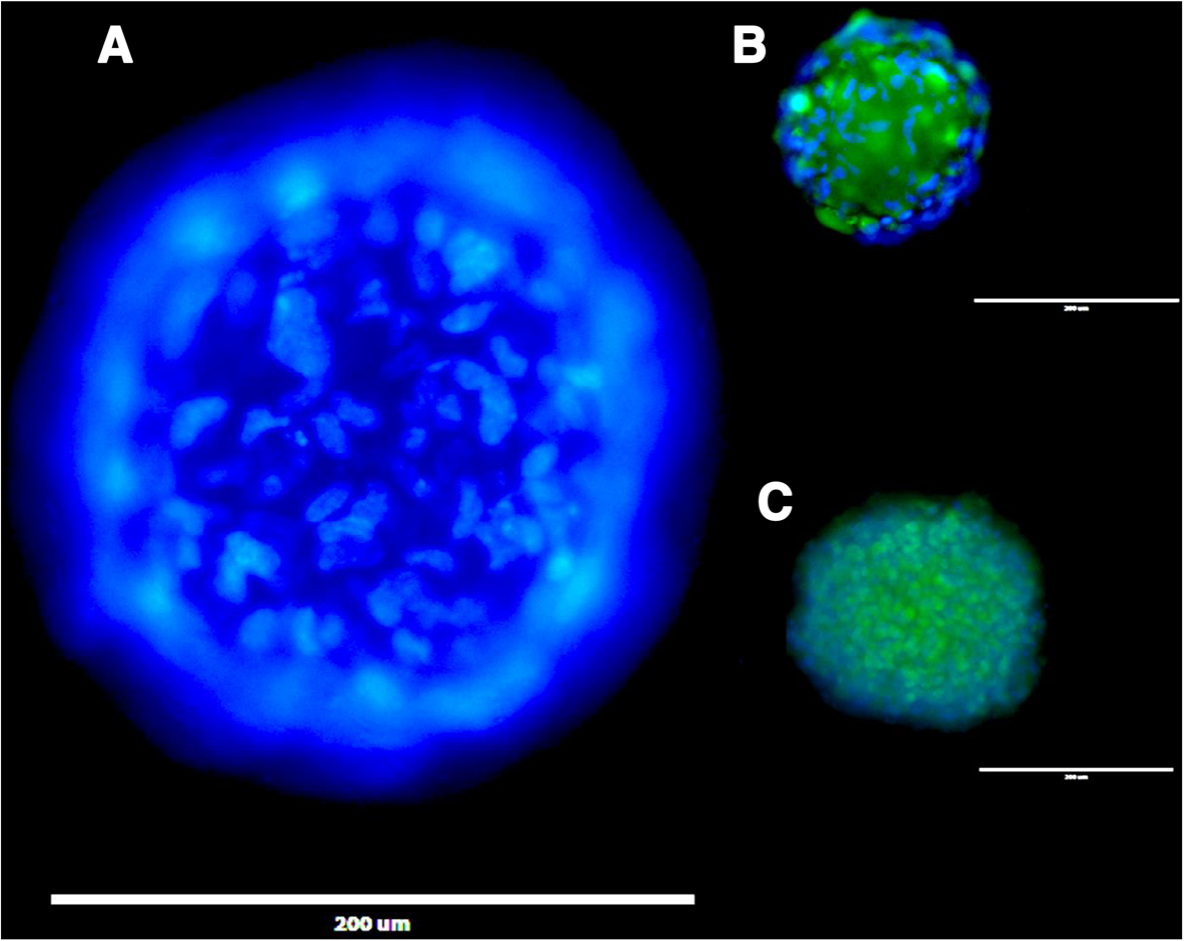
Images of Calcein-AM (green), ethidium homodimer 1 (red), and Hoechst 33342 fluorescence (blue) staining of (**a** and **b**) h-PMSC and (**c**) h-ISMC at 72 h, Scale bar = 200 μm for each (**a**-**c**)

**Fig. 5:**
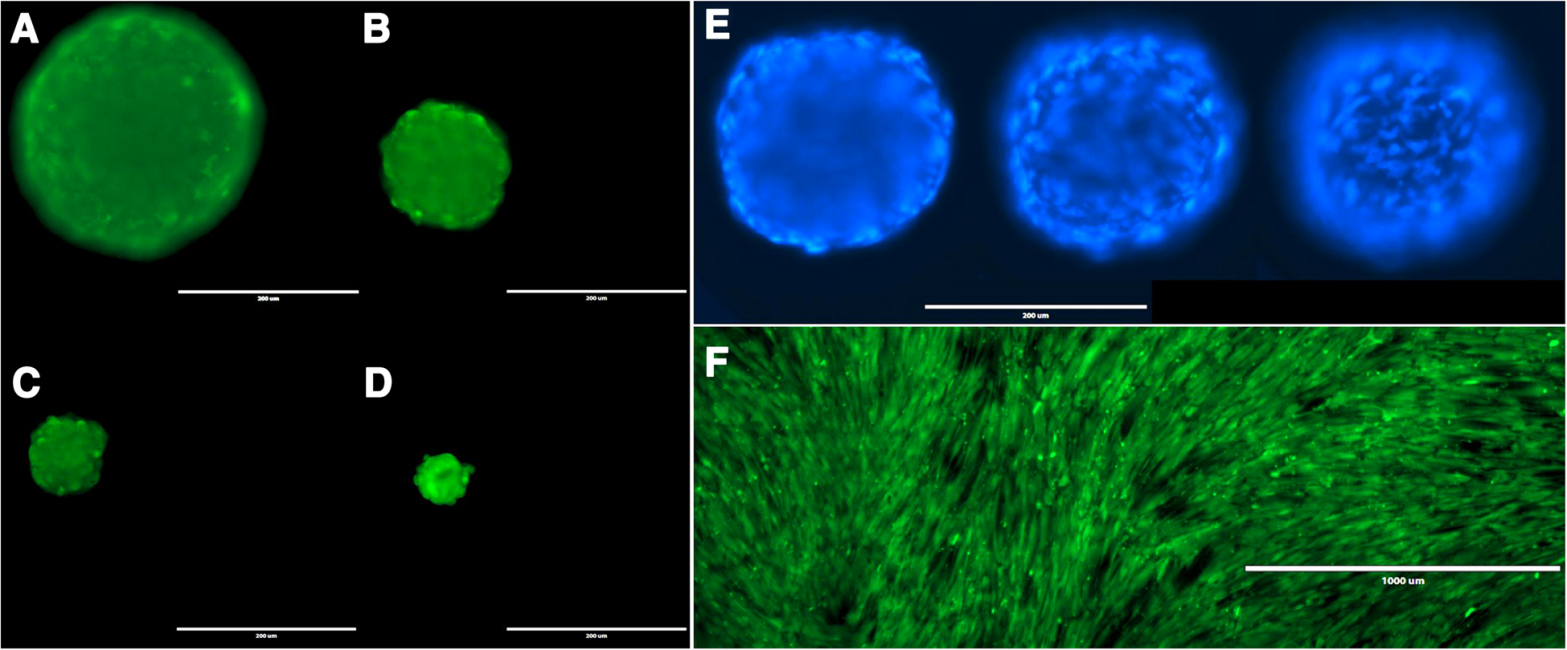
Images of h-PMSC (**a**-**d**) stained with Calein AM at different cell densities at 72 h, scale bars = 200 μm. Images of Hoechst 33342 fluorescence staining of h-PMSC (**e**) at 72 h at different focal planes, scale bar = 200 μm. Image of h-PMSC (**f**) monolayer on a flat polystyrene plate stained with Calein AM, scale bar = 1000 μm

**Fig. 6:**
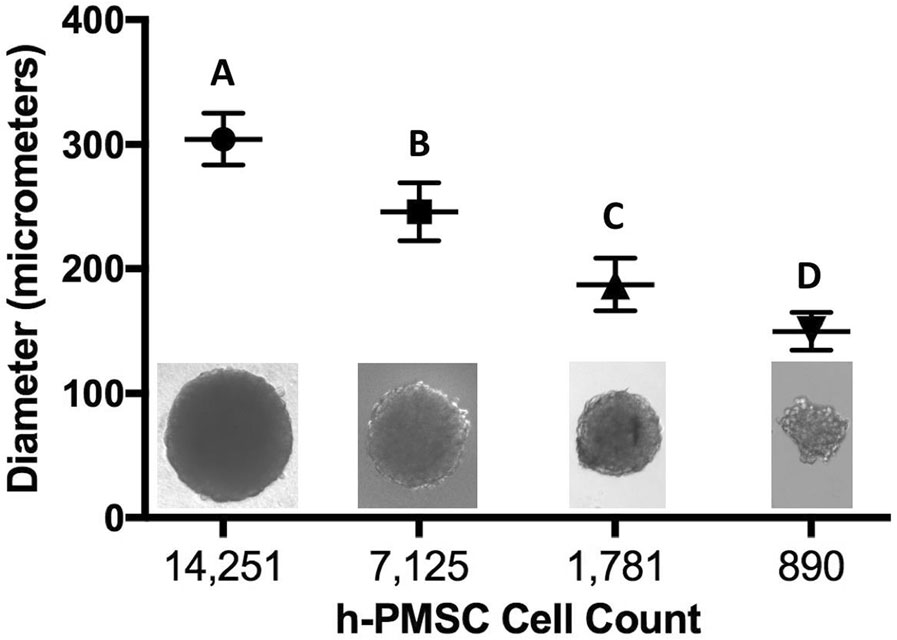
Average diameters of h-PMSC spheroids (**a**-**d**) at different cell densities at 72 h. **a** 14,251 cells, **b** 7,125 cells, **c** 1,781 cells, and **d** 890 cells

The utility of 3D printed inserts as a 3D microtissue generator was confirmed in a set of experiments designed to monitor cellular spheroid formation. 3D printed inserts were capable of maintaining 40 μl of cell-loaded liquid suspensions in 96-well formats (see Figs. 1, 2). Overall, the three different cell types evaluated with the 3D printed insert system were effective in fabricating 3D spheroidal microtissues. Phase microscopy showed progressive formation of spheroids over the course of 72 h (see Fig. 3). At 10 min the U87 cells, showed clear individual cellular dispersal in the liquid suspensions (see Fig. 3a). At 24 h, the U87 cells began to aggregate into multiple cell clusters (see Fig. 3b). At 48 h, the U87 cell clusters had merged into larger clusters (see Fig. 3c). By 72 h, the U87 cells displayed large single spheroidal formations (see Fig. 3d). Similarly, h-PMSC and h-ISMC formed single spheroids by 72 h (see Figs. 4 and 5). Fluorescence staining showed viable live cell clusters for each cell type examined (see Figs. 4 and 5). Cell-density ‘tunability’ of spheroid size was achieved with h-PMSC using different seeding concentrations. This change created spheroids of increasing sized based on the numbers of cells initially injected into the 3D printed inserts and were morphologically different from cellular monolayers (see Fig. 5a and f). Using an *n* = 12 for each dilution set, each dilution series was significantly different in size from each other group (***-*p* < 0.001). Inserts seeded with 14,251 cells had a mean diameter of 304.293 ± 20.8 μm (Mean ± standard deviation (SD)) (see Fig. 6a). Inserts seeded with 7,125 cells displayed a mean diameter of 245.781 ± 23.236 μm (see Fig. 6b). Inserts seeded with 1,781 cells displayed a mean diameter of 187.307 ± 21.298 μm (see Fig. 6c). Inserts seeded with 890 cells displayed a mean diameter of 149.83 ± 15.01 μm (see Fig. 6d). h-PMSCs seeded above 1,781 cells appeared more symmetrical and tight spheroid formations, while h-PMSCs seeded under 890 cells formed less symmetrical and tightly formed spheroids.

## Discussion

Advances in tissue engineering for both physiologic and diseased tissue models have been achieved through 3D printing of tissue scaffolds and direct bioprinting of cells and tissue constructs, both of which have been previous performed with spheroid and tissue-on-a-chip models [15–25]. Although the use of 3D printing and bioprinting has not been fully optimized, promising studies have demonstrated its utility in fabricating implants in humans, tissue-like constructs in animal models, and human-like tissue models for drug screening [17–23]. 3D printed ovary-like constructs have been implanted in mice with surgically removed ovaries. The 3D printed ovary-like constructs had a porous morphology, which accommodated ovarian follicles in various stages of maturity. These bioengineered ovary-like constructs allowed some mice to become impregnated and produce offspring. Several investigators have used bioprinting to engineer tissue constructs for drug screening and disease modeling [25–27]. One group used bioprinting technology to assemble human HepG2/C3A spheroids on a liver-on-a-chip platform and demonstrated feasibility for this model for use in drug toxicity screening [25].

This 3D printed microtissue insert approach can be easily adapted for embryo culture, and in development of tumor models and disease modeling. A potential application of this model is in modeling tumor-endothelial interactions in cancer invasion and metastasis. After droplets containing spheroids have been transferred to well bottoms, the spheroids contact, adhere, and eventually begin to migrate on the polystyrene surfaces. The addition of a specific type of cell layer cell type on underlying well bottom would permit evaluation of adhesive and motile responses in tumor spheroids and their responses to different drug treatments. These considerations are now being explored and will be reported in future studies.

Simple hanging drop concepts, hydrogels, and biomaterials have been devised previously, but are not designed for consumer grade 3D printers in 96-well plate formats [28–30]. Hanging drop style 96-well plates and ultra-low attachment systems exist on the market, but may be challenging for groups to acquire due to the cost. By comparison, the material cost for one PLA 96-well insert described in this study was $0.27 cents ($USD). This translates to $1.08 in material cost for 384 spheroid assays. The 3D printed PLA inserts can be re-sterilized using gamma irradiation or as shown here, using 70% ethanol, which further enhances cost effectiveness. Other high-temperature performing materials, such as polycarbonates can also be used, which would permit autoclaving. These types of customized cell culture inserts therefore have major advantages for research groups with limited funding and access to consumer or commercial grade 3D printers. 3D printing represents an economical and practical tool for ad hoc, de novo, or template-based creation of 3D printed constructs to aid with tissue engineering, cell cultures, and other laboratory experiments [31].

This approach allowed rapid, high throughput and reproducible production of cell spheroids for use in bioactive screening assays. Through this method, a variety of spheroids and co-cultures may be fabricated for personalized medicine research. Higher cell numbers appear to encourage tighter cell-cell binding in spheroids based on smoother profiles; this may be important in models considering surface area, drug penetration and nutrient/oxygen and waste exchange, all of which can be ‘tuned’ using applied cell counts. In this method, 40 μl of cell-media was applied to each insert and cells allowed to grow for 72 h. For testing drugs or bioactive materials against spheroids, an additional 1–15 μl of a desired bioactive-loaded solution can be loaded without compromising drop stability. This system is not limited to scaffold-free cultures, as other biomaterials and precious cargoes (e.g. micro and nanoparticles) may also be added to the suspension cultures for tissue engineering and drug carrier targeting studies. A limitation to the 3D printed microtissue insert is the potential for suspension dehydration overtime. However, 3–5 days is sufficient for spheroid formation in an 100% humidity environment, which is critical for preventing suspension dehydration. For long-term liquid suspension studies, inserts may be modified to house a reservoir of media or liquid to prevent evaporation. Such systems are currently being designed and will be examined in the future.

## Conclusions

The 3D printed microtissue inserts described in the present study represents a cost-effective approach that can be integrated in laboratories even with consumer-grade 3D printers. A variety of tunable 3D spheroid microtissues can be evaluated with this 3D printed insert. Overall, it is suggested that these 3D printed microtissue inserts have potential applications in a variety of drug-delivery, disease modeling, and tissue engineering systems.

## Abbreviations

$USD: United States Dollar
2D: Two-dimensional
3D: Three-dimensional
CAD: Computer-aided design
CAM: Computer-aided modeling
CO_2_: Carbon dioxide
DMEM: Dulbecco’s Modified Eagle’s medium
FBS: Fetal bovine serum
h-ISMC: Human intestinal smooth muscle cells
h-PMSC: Human placental-derived mesenchymal stem cells
hrs.: hours
P/S: Penicillin/streptomycin
PLA: Polylactic Acid
SD: Standard deviation
U87: U87 MG human glioblastoma cells
μl: Microliters
